# Supplementary material for: Region-Specific Gut Microbiome Variation Between Changle Geese and Yellow-Feathered Broilers: Correlations with Growth and Intestinal Development
Source: Microorganisms. 2025 Sep 13;13(9):2145. doi: 10.3390/microorganisms13092145 (PMC12472417; doi:10.3390/microorganisms13092145)
Supplement: Supplementary file 1 [file microorganisms-13-02145-s001.zip › microorganisms-3824522-supplementary.pdf]

# Supplementary Files

## Supplementary Figures

**Figure S1**. The Venn plot of chickens and geese gastrointestinal microbiota on ASV level. Host symbols: c= chicken, g= goose; Intestinal segments: Cr= crop, P= proventriculus, G= gizzard, J= jejunum, Ce= cecum, Cl= rectum. Format: Labels follow the pattern [Host]_[Segment] (e.g., c_Cr= chicken crop; g_Ce= goose cecum).

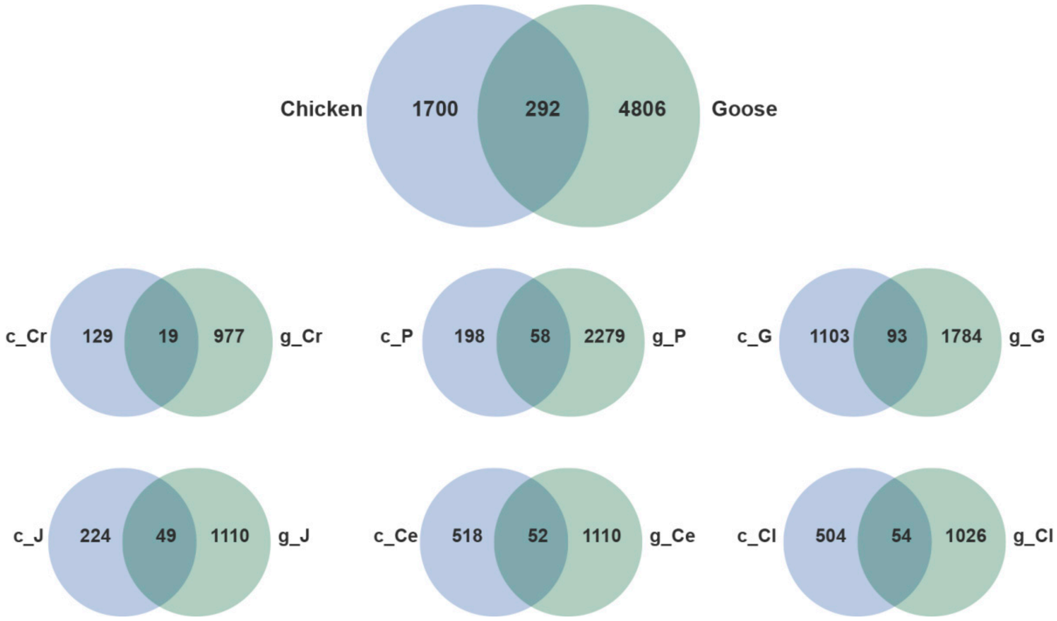

**Figure S2.** The core ASVs in the cecum and rectum between chickens and geese based on random forestry algorithm.

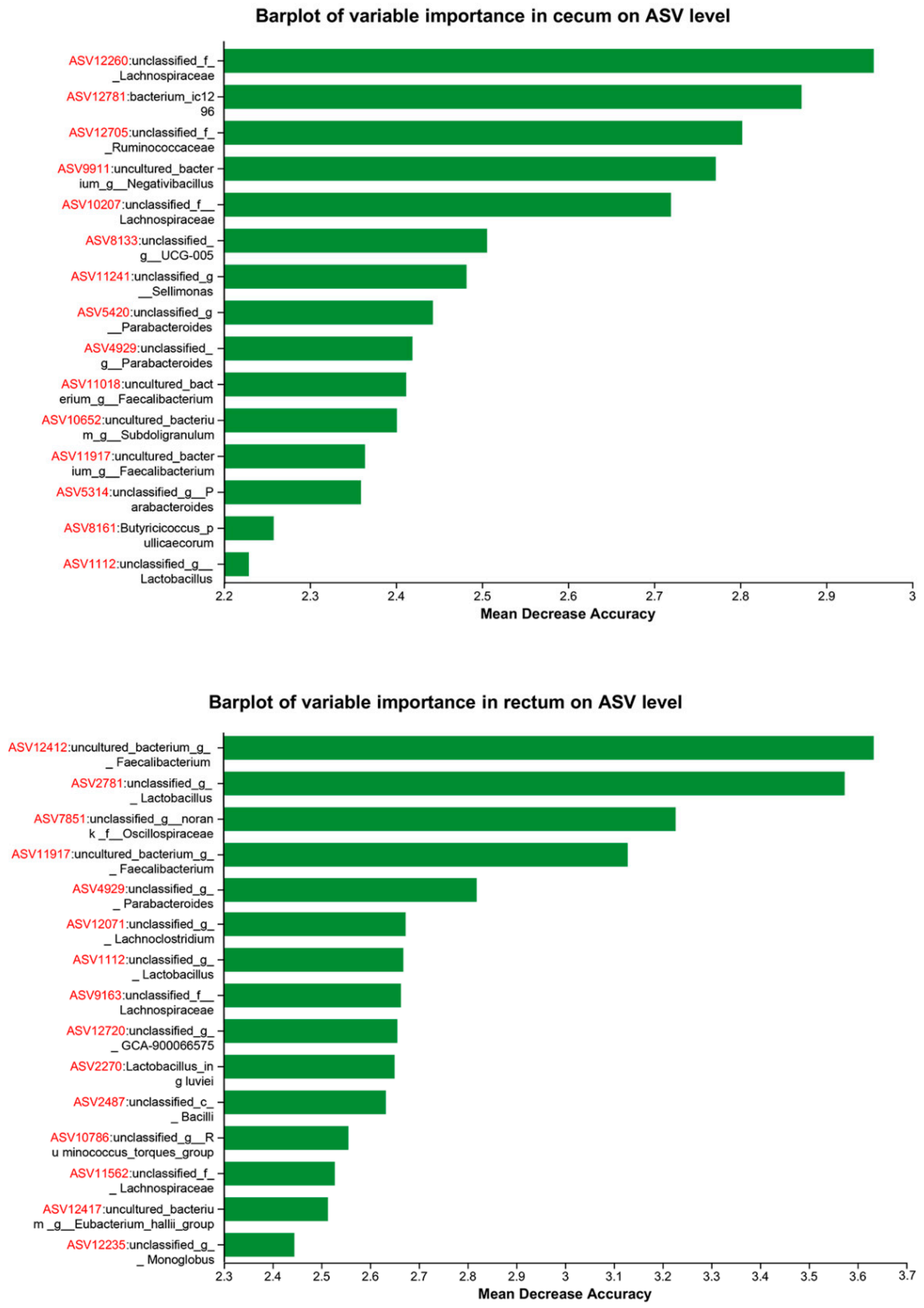

**Supplementary Tables**

**Table S1**. Basal diet composition and nutrient levels for chickens.

| **Items** | **Phase 1** (d 1 to d 21) | **Phase 2** (d 22 to d 42) | **Phase 3** (d 43 to d 63) |
|---|---|---|---|
| Ingredients, % | | | |
| Corn | 58.61 | 63.94 | 72.85 |
| Soybean meal, 46% CP | 27.80 | 21.92 | 13.30 |
| Expanded soybean | 9.00 | 10.00 | 10.00 |
| Limestone powder | 1.24 | 1.05 | 1.00 |
| Dicalcium phosphate | 1.89 | 1.69 | 1.41 |
| DL-Methionine, 98% | 0.16 | 0.10 | 0.07 |
| Lysine | 0.00 | 0.00 | 0.07 |
| Premix[1] | 1.00 | 1.00 | 1.00 |
| NaCl | 0.30 | 0.30 | 0.30 |
| Total | 100.00 | 100.00 | 100.00 |
| Nutrient levels[2] | | | |
| Metabolizable energy, MJ/kg | 12.13 | 12.42 | 12.76 |
| Crude protein, % | 20.83 | 19.00 | 15.99 |
| Calcium, % | 1.00 | 0.87 | 0.77 |
| Total phosphorus, % | 0.68 | 0.63 | 0.55 |
| Non-phytate phosphorus, % | 0.45 | 0.42 | 0.37 |
| Lysine, % | 1.10 | 0.98 | 0.82 |
| Methionine + Cystine, % | 0.85 | 0.75 | 0.64 |

[1] Nutrient levels of premix in Phase 1 (per kg diet): vitamin A, 8000 IU; vitamin $D_3$, 2000 IU; vitamin E, 20 mg; vitamin $K_3$, 1mg; vitamin $B_1$, 2.6 mg; vitamin $B_2$, 5.4 mg; vitamin $B_6$, 5 mg; vitamin $B_{12}$, 0.02mg; nicotinic acid, 40 mg; pantothenic acid, 20 mg; biotin, 0.2 mg; folic acid, 0.8 mg; choline chloride, 1000 mg; Cu (sulfate), 8 mg; Fe (sulfate), 80 mg; Mn (sulfate), 80 mg; Zn (sulfate), 60 mg; I (iodide), 0.35 mg; Se (selenite), 0.15 mg.

Nutrient levels of premix in Phase 2 (per kg diet): vitamin A, 8000 IU; vitamin $D_3$, 2000 IU; vitamin E, 20 mg; vitamin $K_3$, 1 mg; vitamin $B_1$, 2.6 mg; vitamin $B_2$, 5.4 mg; vitamin $B_6$, 5 mg; vitamin $B_{12}$, 0.02mg; nicotinic acid, 35 mg; pantothenic acid, 20 mg; biotin, 0.2 mg; folic acid, 0.8 mg; choline chloride, 750 mg; Cu (sulfate), 8 mg; Fe (sulfate), 80 mg; Mn (sulfate), 80 mg; Zn (sulfate), 60 mg; I (iodide), 0.35 mg; Se (sulfate), 0.15 mg.

Nutrient levels of premix in Phase 3 (per kg diet): vitamin A, 8000 IU; vitamin $D_3$, 2000 IU; vitamin E, 20 mg; vitamin $K_3$, 1 mg; vitamin $B_1$, 2.6 mg; vitamin $B_2$, 5.4 mg; vitamin $B_6$, 5 mg; vitamin $B_{12}$, 0.02mg; nicotinic acid, 30 mg; pantothenic acid, 20 mg; biotin, 0.2 mg; folic acid, 0.8 mg; choline chloride, 500mg; Cu (sulfate), 8 mg; Fe (sulfate), 80 mg; Mn (sulfate), 80 mg; Zn (sulfate), 60 mg; I (iodide), 0.35 mg; Se (Sodium selenite), 0.15 mg.

[2] Nutrient levels are calculated values.

**Table S2**. Basal diet composition and nutrient levels for geese.

| Items | Phase 1 (d 0 to d 28) | Phase 2 (d 29 to d 70) |
|---|---|---|
| Ingredients, % | | |
| Corn | 59.10 | 60.20 |
| Soybean meal | 30.20 | 23.70 |
| Rice hull | 3.10 | 8.50 |
| Wheat bran | 4.80 | 4.70 |
| Salt | 0.30 | 0.30 |
| DL-Methionine | 0.10 | 0.10 |
| Calcium hydrogen phosphate | 1.40 | 1.50 |
| Premix[1] | 1.00 | 1.00 |
| Total | 100.00 | 100.00 |
| Nutrient levels[2] | | |
| Metabolizable energy, MJ/kg | 11.32 | 11.06 |
| Crude protein, % | 19.04 | 16.51 |
| Crude fiber, % | 4.54 | 6.42 |
| Calcium, % | 0.83 | 0.82 |
| Total Phosphorous, % | 0.71 | 0.71 |
| Lysine, % | 0.38 | 0.34 |
| Methionine, % | 0.97 | 0.80 |

[1] Premix provides the following per kilogram of diet：vitamin A, 1500 IU; vitamin $D_3$, 200 IU; vitamin E, 18.5 IU; vitamin $K_3$, 1.5 mg; vitamin $B_1$, 2.2 mg; vitamin $B_2$, 5 mg; vitamin $B_6$, 2 mg; vitamin $B_{12}$, 0.01 mg; nicotinic acid, 65 mg; pantothenic acid, 15 mg; biotin, 0.2 mg; folic acid, 0.5 mg; choline chloride, 1000 mg; Cu (sulfate), 50 mg; Fe (sulfate), 85 mg; Mn (sulfate), 80 mg; Zn (sulfate), 80 mg; I (iodide), 0.42 mg; Se (Sodium selenite), 0.3 mg.

[2] Nutrient levels are calculated values.
